# A Case of Delayed Cecal Perforation After Abdominal (Seat Belt) Injury

**DOI:** 10.7759/cureus.27901

**Published:** 2022-08-11

**Authors:** Raymond I Okeke, Justin Lok, Prajwal Keranalli, Maaria Chaudhry, Christian Saliba, Richard Herman, L R Tres Scherer, Shin Miyata, Christopher Blewett

**Affiliations:** 1 General Surgery, Saint Louis University School of Medicine, Saint Louis, USA; 2 Pediatric Surgery, SSM Health Cardinal Glennon Children's Hospital, Saint Louis, USA

**Keywords:** seatbelt, bowel injury, seatbelt syndrome, perforation, bowel

## Abstract

Seatbelts have reduced the number of fatal head, facial, and chest injuries. They have, however, introduced a set of injuries comprising abdominal wall bruising, Intra-abdominal injuries, and lumbar spine fractures collectively termed the seat belt syndrome. Surgical repair is the treatment for encountered bowel injuries. We present a case of delayed bowel perforation following presentation with signs of seat belt trauma identifying a decisional dilemma in the surgical management of serosal tears with no apparent signs of perforation.

## Introduction

Thirty million children under 18 years of age visit the emergency department annually. Of these cases, 7.5 million visits are due to trauma. Most blunt injuries are due to falls and motor vehicle accidents [1]. In 2020, there were five million reported car crashes in the United States [2]. Seatbelts, specifically three-point seatbelt systems that cover both chest and lap, have reduced mortality by 45% and the risk of serious injury by 50% [2]. Despite these protective qualities, seat belts have introduced a set of injuries called seat belt syndrome [3]. First described in 1962, it includes a constellation of injuries such as lumbar spine fractures (L2-L4), abdominal wall bruising, and intra-abdominal injuries to solid and hollow organs [3] with colonic injuries comprising 0.5% of identified injuries [4]. When hollow viscus injuries fail conservative management, surgical intervention is needed. We present a case of delayed cecal perforation in a nine-year-old female after a motor vehicle accident highlighting the importance of a high index of suspicion in preventing delay in appropriate care.

## Case presentation

**Figure 1:**
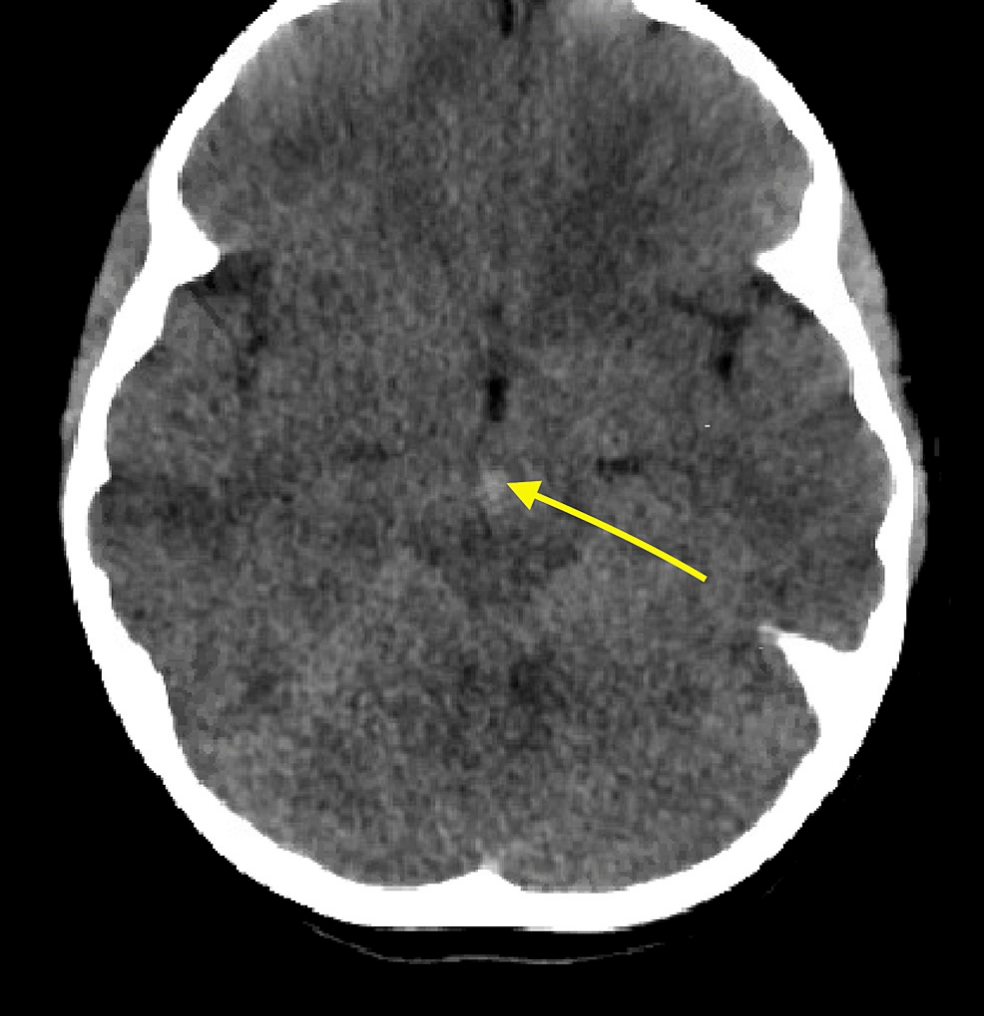
Subarachnoid hemorrhage in the subarachnoid cisterns (arrow).

**Figure 2:**
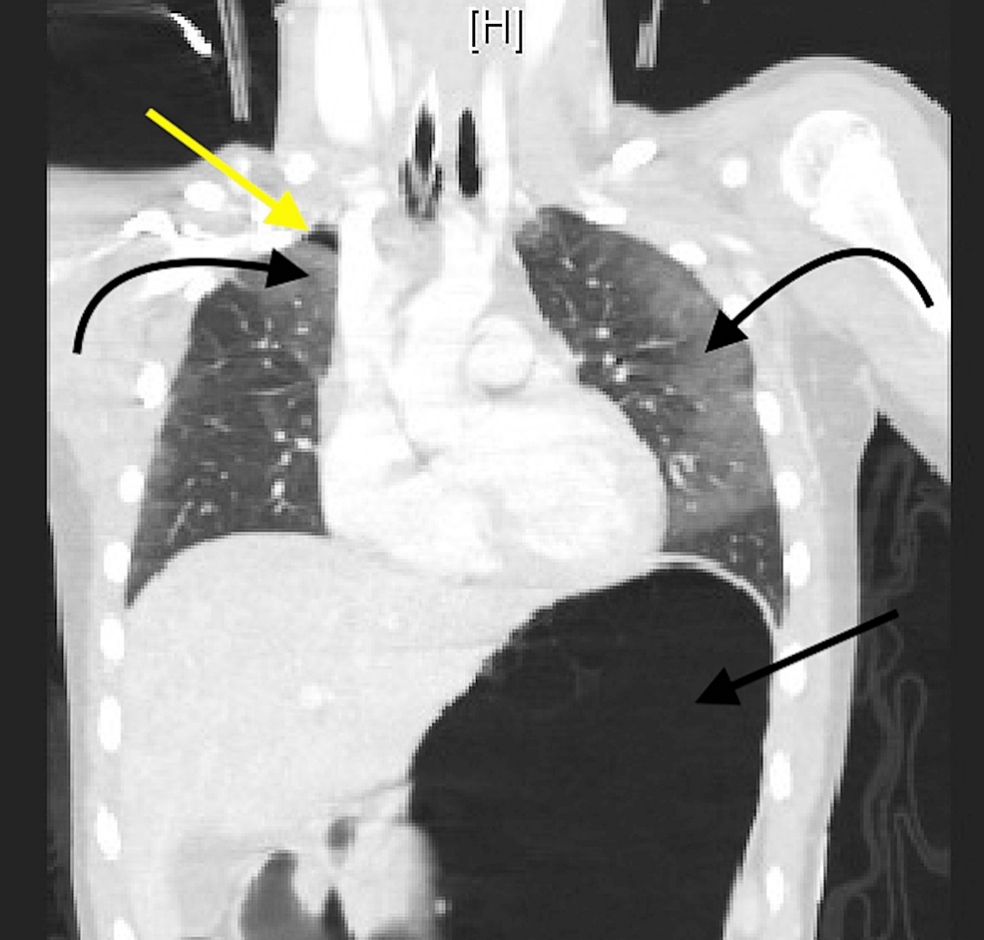
CT chest and abdomen showing right apical pneumatocele (yellow arrow), bilateral pulmonary contusions (curved black arrows), and large stomach (straight black arrow).

EMS brought in a nine-year-old female after a head-on motor vehicle collision going 45-50 miles per hour in which she was a back seat restrained passenger. The patient was intubated at the scene for a Glasgow Coma Scale (GCS) of 3 and arrived with a cervical collar in place. The primary survey revealed seat belt abrasions and ecchymoses across her chest and bilateral hips. She also bruised her forehead, left upper back, and left anterior thigh. Her abdomen was minimally distended and non-rigid. Computed tomography (CT) of the head and spine showed a subarachnoid hemorrhage around the basal cisterns to the level of cervical spine C1-C2, cerebral edema, and no spinal injuries (Figure 1). A chest, abdomen, and pelvis (CAP) CT scan revealed a 0.6x1 cm right apical pneumatocele, bilateral pulmonary contusions, and some free fluid in the abdomen (Figure 2).

**Figure 3:**
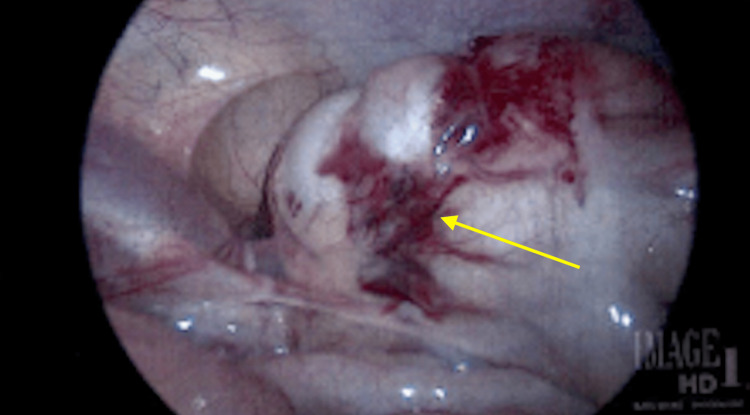
Serosal tear to cecum identified on diagnostic laparoscopy (arrow).

**Figure 4:**
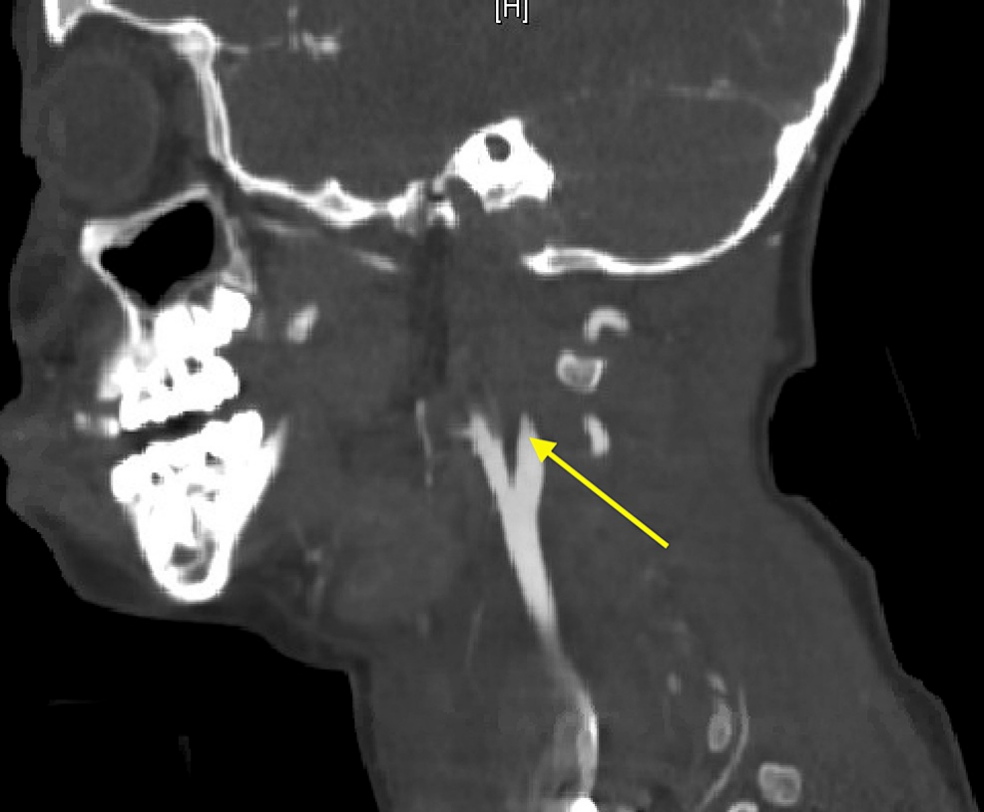
Occlusive right internal carotid dissection (arrow).

We performed a diagnostic laparoscopy given concern for intra-abdominal injury with seat belt sign, revealing a cecal serosal tear that did not compromise the cecal wall integrity (Figure 3). With no frank perforation, the surgical team decided not to suture across the serosal tear. The patient started aspirin on postoperative day (POD) zero for an occlusive right internal carotid artery dissection identified on a CT angiogram of the neck (Figure 4). In addition, she required intra-cranial pressure (ICP) monitoring and hypertonic saline for increased ICPs for the next seven days. She also underwent occiput to C2 posterior spinal instrumented fusion for cervical occipital dissociation on presentation.

On postoperative day (POD) nine, the patient became febrile to 101.8°F with increasing abdominal distension. CT imaging obtained revealed pneumoperitoneum (Figure 6). We performed an exploratory laparotomy which identified a cecal perforation in the region of the serosal tear with gross spillage necessitating an ileocecectomy. We did not identify any other injuries and had no concerns for intra-abdominal vascular compromise. After abdominal washout, we created an ileostomy and a mucous fistula from the ascending colon. Postoperatively, the patient had a return of bowel function on POD one, was extubated on POD four, and tolerated enteral feeds by POD seven. Broad spectrum antibiotics were continued postoperatively, then de-escalated and discontinued at discharge to a long-term acute care facility on POD 14.

**Figure 5:**
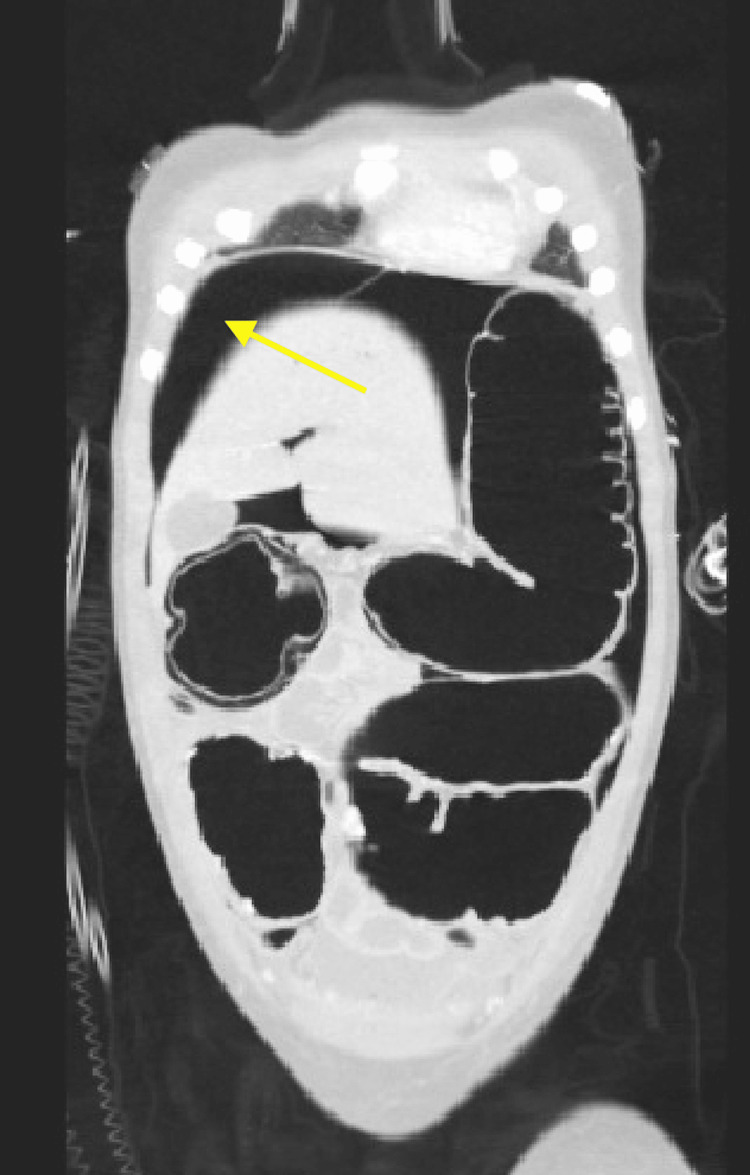
Pneumoperitoneum to right upper quadrant (arrow).

At the one-month clinic follow-up, the patient had been discharged home from the long-term acute care facility. She has had neurologic recovery with no sensory deficits but continues to need occupational and speech therapy for reconditioning. She continues to tolerate a regular diet and ambulates at home. She has a wheelchair for longer distances. She subsequently underwent ileostomy reversal with no complications and continues to do well.

## Discussion

Seat belts were introduced in the 1960s and have reduced motor vehicle collision fatalities. In 1960, the Department of Transportation data reported 5.06 deaths per 100 million vehicle miles, dropping to 1.34 per 100 million vehicle miles in 2010 [2]. In addition, the introduction of seatbelts decreased the number of fatal head and chest injuries [3] but introduced a trifecta of injuries that make up seatbelt syndrome. These include lumbar fractures, abdominal wall bruising, and intra-abdominal injuries, including solid and hollow [4,5]. A study on seatbelt syndrome in Canadian children reported that one in 1000 children in motor vehicle collisions presented with seatbelt syndrome [6]. Pediatric patients are at higher risk of seatbelt syndrome as they have thinner abdominal walls, making them more susceptible to blunt force trauma leading to hollow viscus injuries. Motor vehicle accidents are the most common cause of blunt trauma colon injuries [1,7]. Injuries to the transverse colon are the most frequent, with sigmoid and ascending colon injuries less prevalent [7,8].

Blunt trauma colon injury can occur from crushing the colonic segment between the seat belt and vertebrae or pelvis [7]. Rapid deceleration can result in shearing between the mobile regions of the colon and the natural fixed points at the sigmoid and ileocecal regions, causing sigmoid and ascending colon injury [7,8]. Colonic burst injuries can also occur secondary to mechanical occlusion during blunt trauma when intra-luminal pressure exceeds the colonic wall’s tensile strength [8,9]. Common blunt trauma colon injury patterns include serosal tears, contusions, laceration, transection, mesenteric tear, and hematoma [9,10]. Colonic devitalization secondary to devascularization causes delayed colonic perforation [8]. Blunt trauma colon injuries can occur in tandem with small bowel, liver, spleen, or kidney injuries [7]. Isolated blunt trauma colonic injuries are rare [4,11]. We recommend a combination of serial abdominal exams, x-ray, ultrasound, and computed tomography scans as indicated for diagnostic workup [7-9].

The absence of clear signs like peritonitis and pneumoperitoneum does not rule out blunt trauma colon injury, so we recommend a high index of suspicion [8,9]. To intervene in blunt trauma abdominal injuries, surgeons perform exploratory laparotomies [12]. Diagnostic laparoscopy reduces the incidence of negative laparotomies and the length of hospital stay in the hemodynamically stable patient with concern for blunt trauma colon injury [13,14]. Our patient presented with neurologic injury and thus was not hemodynamically stable on presentation and may have benefitted from an initial exploratory laparotomy. Primary closure, resection with anastomosis, or colostomy are treatment options depending on the size of the colonic injury, gross contamination, and signs of ischemia or bowel necrosis [7-9]. The repair of serosal tears, when encountered especially with no signs of further devastation or mesenteric injury, has been questioned in the literature [15]. We, however, recommend considering exploratory laparotomy in cases like ours with an intubated patient, other injuries, and concern for abdominal injury. A laparotomy should be the next step should a diagnostic laparoscopy shows any evidence of injury, including serosal tears. We suspect local colonic devitalization caused the delayed cecal perforation, although we did not note any signs of mesenteric injury or ischemic bowel at the time of laparoscopy or subsequent laparotomy.

Preventing sepsis from colonic perforation outweighs the risk of a negative laparotomy in intubated trauma patients with any evidence of large bowel compromise [16]. Since these patients are unable to participate in serial abdominal examinations, prompt intervention and treatment are warranted.

## Conclusions

We corroborate findings in the literature that a high index of suspicion for bowel injury is necessary for motor vehicle accidents with signs of seat belt trauma. We add that the benefits of preventing adverse sequelae from a delayed bowel perforation outweigh the risk of a negative laparotomy in neurologically compromised trauma patients with any evidence of bowel injury. Serosal tears with diagnostic laparoscopy if performed should necessitate an immediate exploratory laparotomy in this trauma population.
